# Five unaddressed questions about cytokinin biosynthesis

**DOI:** 10.1093/jxb/erae348

**Published:** 2024-09-19

**Authors:** Hitoshi Sakakibara

**Affiliations:** Graduate School of Bioagricultural Sciences, Nagoya University, Nagoya, Japan; RIKEN Center for Sustainable Resource Science, 1-7-22, Suehiro, Tsurumi, Yokohama, Japan; University of Sydney, Australia

**Keywords:** *Agrobacterium tumefaciens*, *Arabidopsis thaliana*, biosynthesis, *cis-*zeatin, crown gall, cytokinin, metabolism, *Oryza sativa*, plant hormone, *trans-*zeatin

## Abstract

Cytokinins, a class of phytohormones, play crucial roles in regulating plant growth and stress responses through finely tuned feedback loops involving metabolic and signaling cascades. Over the past 25 years, studies have identified key genes involved in cytokinin biosynthesis and inactivation pathways. Nevertheless, several gaps remain in our understanding, particularly regarding the movement of intermediate metabolites between subcellular compartments and the discrepancy between the products of adenosine phosphate-isopentenyltransferase (IPT) and the substrate preferences of subsequent reactions. Recent gene discoveries related to lonely guy (LOG)-independent pathways suggest a spatial extension of cytokinin biosynthesis into the apoplast. Other intriguing issues remain to be addressed, such as elucidating the synthetic pathway for *cis-*zeatin and unraveling the molecular mechanisms governing selective substrate use by the cytokinin biosynthetic enzyme Tumor morphology root (Tmr) from the phytopathogen *Agrobacterium tumefaciens*. Further studies are needed to reveal a fully comprehensive picture of cytokinin metabolism.

## Teaser

Although the basic framework for cytokinin biosynthesis has been elucidated, revisiting enzyme cellular and subcellular localizations and substrate specificities suggests that genes involved in several key processes may remain uncharacterized.

## Introduction

Phytohormones serve as signaling molecules in multiple aspects of plant growth and environmental stress responses. Their actions are regulated by both metabolic and signaling systems that operate under a tightly controlled feedback loop. The metabolic systems primarily determine the abundance of biologically active hormone molecules. Biosynthesis can be likened to a main valve that controls the production of active hormone molecules, whereas inactivation acts as a drain, regulating the accumulation level within an optimal range. Modulation of the inactivation process appears to be critical for maintaining desirable hormone levels. Plants often employ multiple inactivation pathways, including irreversible conjugation, degradation, and oxidation, as well as reversible processes such as glycosylation and amino acid conjugation (Osugi and Sakakibara, 2015; Chen *et al.*, 2020, 2021; Hedden, 2020; Casanova-Sáez *et al.*, 2021; Hayashi *et al.*, 2021). In addition, hormone action can be modulated by storage sequestration in intracellular compartments such as the endoplasmic reticulum (ER) or vacuoles (Zhang *et al.*, 2023).

**Fig. 1.**
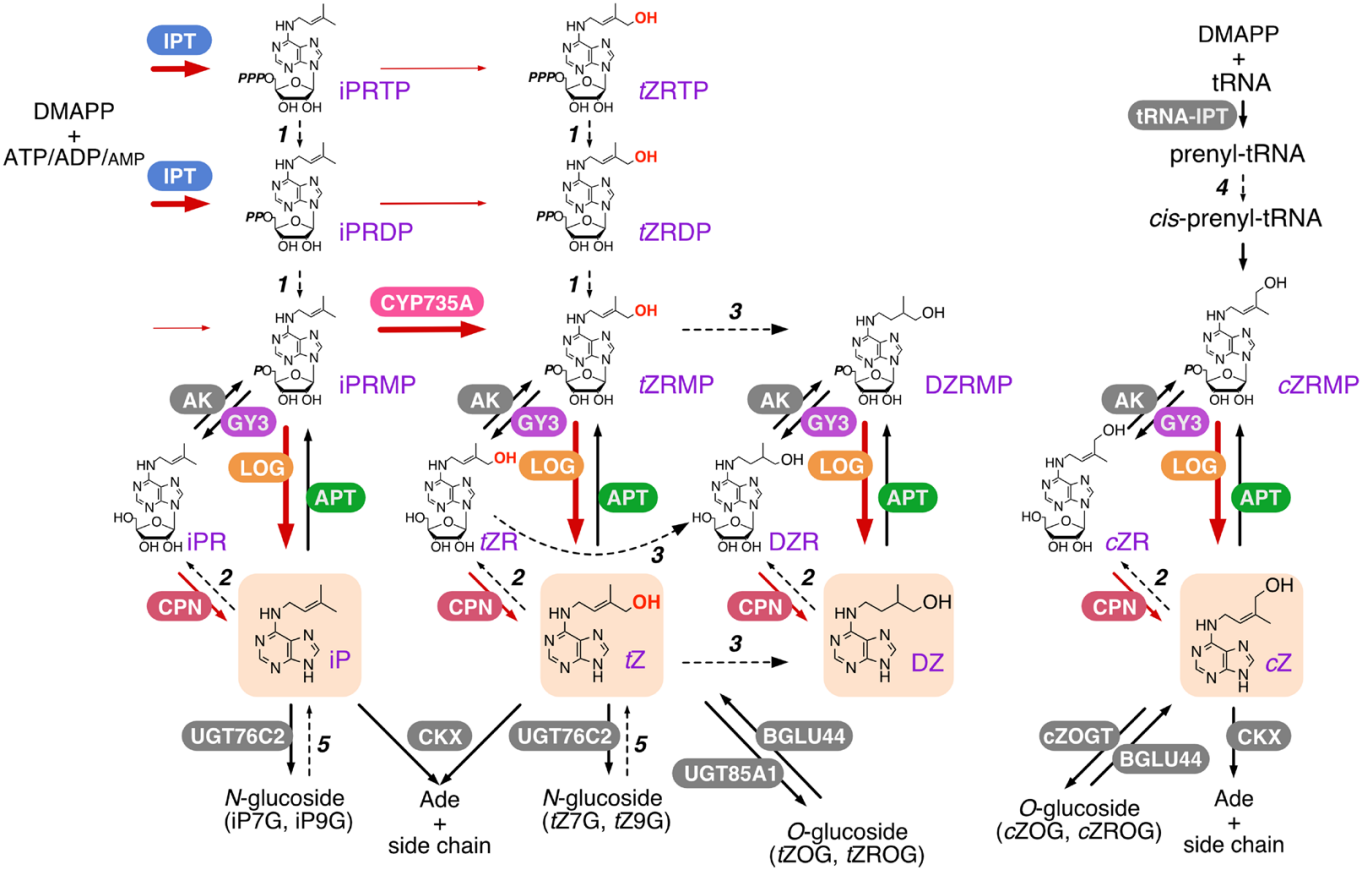
Current model of isoprenoid cytokinin biosynthesis pathways in plants. Plant adenosine phosphate-isopentenyltransferase (IPT; blue) preferably uses ATP or ADP as isoprenoid acceptors and DMAPP as the donor to form iPRTP and iPRDP, respectively. Dephosphorylation of iPRTP and iPRDP by phosphatase (*1*), phosphorylation of iPR by adenosine kinase (AK; grey), and conjugation of phosphoribosyl moieties to iP by adenine phosphoribosyltransferase (APT; green) create the metabolic pool of iPRMP. The iP nucleotides are converted into the corresponding *t*Z-nucleotides by CYP735A (cytochrome P450 monooxygenase 735A; pink). Cytokinin nucleoside 5'-monophospates, such as iPRMP, *t*ZRMP, DZRMP, and *c*ZRMP, are activated to cytokinin nucleobases by a one-step reaction catalyzed by lonely guy (LOG; orange) or by a two-step reaction catalyzed by 5'-ribonucleotide phosphohydrolase grain yield 3 (GY3; purple) and cytokinin/purine riboside nucleosidase (CPN; dark pink). iP, *t*Z, and their nucleosides can be catabolized by cytokinin oxidase (CKX) to adenine (Ade) or adenosine (Ado). *t*Z can be converted to the *O*-glucoside by *O*-glucosyltransferase UGT85A1 and reactivated by β-glucosidase BGLU44. Cytokinin nucleobases also can be converted to the *N*-glucoside by *N*-glucosyltransferase UGT76C2. *2*, purine nucleoside phosphorylase; *3*, zeatin reductase; *4*, *cis*-hydroxylase; *5*, tentatively a β-glucosidase. The width of the red arrows indicates the strength of the metabolic flow discussed in the text. Flows indicated by dashed arrows are not yet well characterized.

One group of phytohormones, the cytokinins, regulate various aspects of plant growth, including leaf senescence, shoot branching, root system development, seed yield and stress responses (Jameson and Song, 2016; Barbier *et al.*, 2019; Cortleven *et al.*, 2019; Svolacchia *et al.*, 2020; Chen *et al.*, 2021; Sakakibara, 2021; Shimadzu *et al.*, 2023). Cytokinins are adenine derivatives with a prenyl side chain at the adenine *N*^*6*^ position, such as *N*^*6*^-(∆^2^-isopentenyl)adenine (iP), *trans*-zeatin (*t*Z), *cis*-zeatin (*c*Z), and dihydrozeatin (DZ) (Fig. 1) (Letham and Palni, 1983; Mok and Mok, 2001; Sakakibara, 2006). They are first formed as nucleotide precursors and then converted to active free bases, and can be cycled back through purine-metabolizing enzymes (Allen *et al.*, 2002; Schoor *et al.*, 2011; Zhang *et al.*, 2013). Previous studies have identified key genes involved in the *de novo* biosynthesis, reversible/irreversible inactivation, and reactivation pathways of cytokinins, thereby establishing a basic framework (Fig. 1) (Letham and Palni, 1983; Mok and Mok, 2001; Sakakibara, 2006; Kieber and Schaller, 2014; Osugi and Sakakibara, 2015; Schaller *et al.*, 2015). Characterization of the enzymes has revealed their substrates, products, and subcellular localizations. Cumulative evidence has shown that these reactions occur in multiple subcellular compartments. In some cases, the products of the preceding reaction and the substrates for the subsequent reaction are not fully matched. This suggests that intermediate metabolites move across membranes between subcellular compartments and that additional enzymes may be involved to fill the gap between products and substrates to complete the series of metabolic processes. These unexplored processes cannot be overlooked in the quest to fully understand cytokinin metabolism, as they may be limiting factors in the biosynthetic flux. In addition, other important issues remain unanswered, such as the identity of a critical reaction in the biosynthesis of *c*Z and the mechanism for selective substrate utilization in cytokinin biosynthesis catalyzed by a phytopathogenic bacterial enzyme. This review will discuss the importance of these processes and potential mechanisms that have received limited attention.

## Question 1. How is the iP nucleotide, the product of IPT, delivered to the cytosol?

**Fig. 2.**
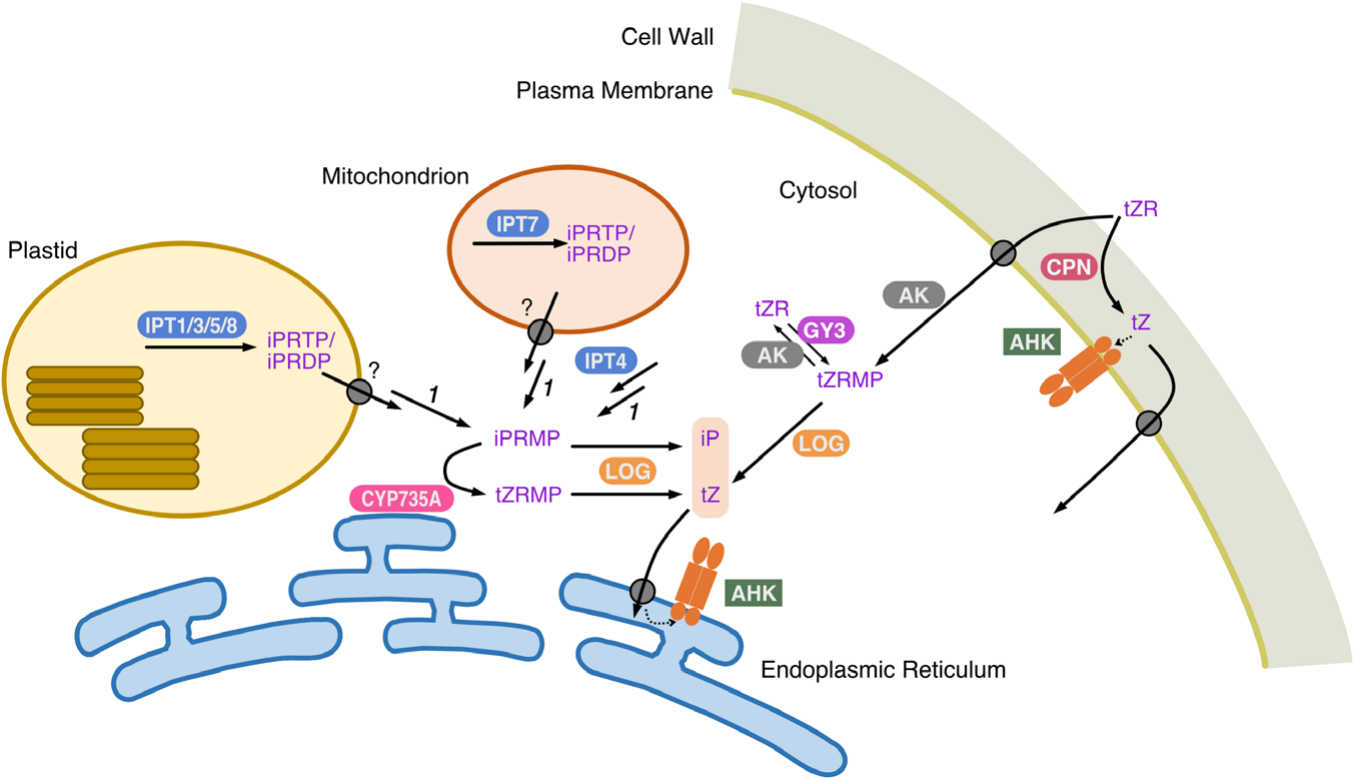
Schematic representation of the metabolic flow of cytokinins at the subcellular level. IPTs (blue) localize in plastids, mitochondria, and the cytosol, and CYP735A (pink) localizes to the endoplasmic reticulum membrane. LOG (orange) localizes in the cytosol. Transport systems for cytokinin precursors to plastids and mitochondria have not been identified. Solid arrows represent metabolic flow, and the dashed line represents recognition by cytokinin sensory histidine kinase AHK. *1*, phosphatase;?, indicates an unidentified transporter; AK, adenosine kinase; CPN, cytokinin/purine riboside nucleosidase; CYP735A, cytochrome P450 monooxygenase 735A; GY3, 5'-ribonucleotide phosphohydrolase grain yield 3; IPT, adenosine phosphate-isopentenyltransferase; LOG, cytokinin riboside 5'-monophosphate phosphoribohydrolase lonely guy.

The initial reaction in cytokinin biosynthesis is catalyzed by adenosine phosphate-isopentenyltransferase (IPT), which conjugates the prenyl-moiety to the *N*^*6*^-position of adenosine phosphate, with dimethylallyl diphosphate (DMAPP) as donor (Fig. 1). A small gene family encodes IPT; the encoded isoenzymes in the eudicot *Arabidopsis thaliana* (Arabidopsis) and the monocot *Oryza sativa* (rice) localize to three subcellular compartments, namely plastids, mitochondria, and cytosol, determined by the expression of a fluorescent translational fusion protein (Kasahara *et al.*, 2004; Kamada-Nobusada *et al.*, 2013). These findings suggest that the IPT reaction occurs in these organelles in a wide variety of plant species (Fig. 2). Based on the expression intensity of the genes and the growth phenotypes observed in their loss-of-function mutants, the plastid-localized types, especially IPT3 and IPT5 in Arabidopsis and OsIPT4 in rice, are thought to serve as major producers of cytokinins (Takei *et al.*, 2004*a*; Miyawaki *et al.*, 2006; Matsumoto-Kitano *et al.*, 2008; Kamada-Nobusada *et al.*, 2013; Ohashi *et al.*, 2017). Due to the relatively high expression level, the mitochondrial IPT, IPT7 in Arabidopsis and OsIPT7 in rice, plays an important role. Analysis of tissue-specific gene expression using a promoter:reporter system showed that *de novo* cytokinin biosynthesis occurs predominantly in non-photosynthetic tissues such as the phloem and pericycle (Miyawaki *et al.*, 2004; Takei *et al.*, 2004*a*; Kamada-Nobusada *et al.*, 2013).

Unlike slime mold and phytopathogen IPTs which use AMP as the prenyl acceptor substrate, vascular plant-type IPTs mainly use ATP or ADP as the substrate, resulting in the production of iP riboside 5'-triphosphate (iPRTP) or iP riboside 5'-diphosphate (iPRDP), respectively (Kakimoto, 2001; Sakano *et al.*, 2004; Sakamoto *et al.*, 2006; Brugière *et al.*, 2008) (Fig. 1). These nucleotides need to co-localize with a cytochrome P450 monooxygenase CYP735A, which is predicted to reside on the ER membrane, or as a lonely guy (LOG) localized in the cytosol. CYP735A adds the hydroxyl moiety to the side chain, producing *t*Z nucleotides, while LOG directly converts nucleotides to the biologically active free bases *t*Z and iP. In order to reach these enzymes, the primary products of IPT have to cross the plastid or mitochondrial envelope membranes (Fig. 2), however the mode of transport remains poorly characterized. *Agrobacterium* IPT Tumor morphology root (Tmr) generates a significant amount of *t*Z riboside 5'-monophosphate (*t*ZRMP) in plastids during crown gall formation upon infection (Palni *et al.*, 1983; Morris, 1986; Sakakibara *et al.*, 2005), but the mechanism responsible for transporting this nucleotide precursor to the cytosol remains unknown. Since nucleotides generally have low membrane permeability, it is reasonable to assume that even with the addition of a prenyl group, their translocation to the cytoplasm by simple diffusion would be highly inefficient. If simple diffusion without a transport system occurs, the membrane permeation step could serve as the primary rate-limiting step in cytokinin biosynthesis. However, it is more likely that nucleotide export from organelles requires the involvement of a transport system.

Although the potential existence of a cytokinin nucleotide precursor-specific transport system should not be disregarded, a structurally homologous system to nucleotide transporters could function on organellar membranes. Mitochondria employ membrane-associated ATP/ADP exchange transporters (AAC1 to AAC3 and related carriers) to deliver ATP to the cytoplasm through an exchange transport with ADP (Haferkamp *et al.*, 2011; Haferkamp and Schmitz-Esser, 2012). iPRTP may be transported to the cytoplasm via an analogous transport system; if iPRDP can be converted to iPRTP through adenine nucleotide metabolism in mitochondria, it is conceivable that the delivery of cytokinin precursors from mitochondria to the cytosol occurs in the form of triphosphates (Fig. 2). On the other hand, ATP in the chloroplast stroma is not transported to the cytoplasm, as the envelope membrane lacks an ATP-permeable system. Triosephosphate, a product of photosynthesis, is transported to the cytoplasm through a triosephosphate/phosphate transporter located on the envelope, serving as an energy source and a building block for synthesizing organic compounds (Rolland *et al.*, 2012). In essence, transported triosephosphate undergoes conversion into sugar that is subsequently transformed into ATP in mitochondria. This pathway is well-characterized in photosynthetic cells, but little is understood about the ATP transport system in plastids of non-photosynthetic cells in which IPTs are predominantly expressed.

NTT1 and NTT2, two Arabidopsis transporters that are responsible for ATP/ADP exchange across the chloroplast inner membrane (Neuhaus *et al.*, 1997; Möhlmann *et al.*, 1998), have been identified, along with AtBt1, a transporter implicated in the efflux of AMP, ADP, and ATP (Kirchberger *et al.*, 2008). Although their ability to transport cytokinin nucleotides has not been confirmed, NTT1 and NTT2 might participate in exporting cytokinin precursors from plastids considering their expression in non-photosynthetic organs such as roots (Reiser *et al.*, 2004; Kirchberger *et al.*, 2008). If NTT1 and NTT2 are involved in cytokinin nucleotide transport, ATP would presumably be taken up by plastids in non-photosynthetic cells, and cytokinin precursors would be released into the cytosol as iPRDP. On the other hand, if AtBt1 functions in their transport, cytokinin nucleotides could be released in any of the three forms (Fig. 2).

Another possibility is the presence of cytokinin nucleotide precursor-specific efflux transporters associated with organelle membranes. Several types of transporters, such as ATP-binding cassette transporter subfamily G14 (ABCG14), AZA-guanine resistance (AZG) AZG1 and AZG2, and purine permease 14 (PUP14), are involved in cytokinin transport, but all types are plasma membrane- or ER membrane-localized (Ko *et al.*, 2014; Zhang *et al.*, 2014; Nedvěd *et al.*, 2021; Tessi *et al.*, 2021, 2023; Xu *et al.*, 2024) (Supplementary Fig. S1). Further exploration, including an investigation of transporters localized on plastids or mitochondria, is necessary to answer how iP is delivered to the cytosol.

## Question 2. How do plants bridge the differences in the number of nucleotide phosphate groups between IPT products and CYP735A and LOG substrates?

As mentioned above, ATP or ADP are substrates for vascular plant-type IPTs, and their products are iPRTP and iPRDP, respectively. NMR analyses of ATP and ADP from cultured cells of sycamore (*Acer pseudoplatanus*) showed a similar abundance of ATP and ADP in plastids and mitochondria (Gout *et al.*, 2014), suggesting that both forms can be used by IPT as substrates *in vivo* as well. Therefore, it is highly likely that both products are supplied to the cytosol from these organelles. On the other hand, iP riboside 5´-monophosphate (iPRMP) is the best substrate for CYP735A. The *k*_cat_/*K*_m_ values for iPRDP and iPRTP are about one third and one hundredth that for iPRMP, respectively (Takei *et al.*, 2004*b*). Also, LOG exclusively uses cytokinin riboside 5'-monophosphate (Kurakawa *et al.*, 2007; Kuroha *et al.*, 2009). Thus, there is a gap in the number of phosphate groups between IPT products and the CYP735A and LOG substrates (Fig. 1). Although there are not many studies on the intracellular ATP, ADP, and AMP quantity ratio, animal cells have been reported to have ratios of ATP:ADP ≈10:1 and ATP:AMP ≈100:1 in the cytosol (Gout *et al.*, 2014; Hardie, 2018). These ratios are regulated by adenylate kinase and ATPase/ATP synthases. If cytokinin precursors can also serve as substrates in these metabolic pathways, the concentrations of iPRTP are maintained at a maximal level. Thus, it is reasonable to assume the occurrence of an enzyme involved in linking IPT with CYP735A and LOG; however, few studies have been focused on this possibility. Given that *Agrobacterium* and slime mold IPTs use AMP as a prenyl-group acceptor substrate (Taya *et al.*, 1978), the enzyme likely emerged in evolutionary coordination with the vascular plant-type IPT.

## Question 3. What is the physiological significance of the LOG-independent pathway?

The conversion of cytokinin nucleotide precursors into their active forms is predominantly mediated by LOG, a phosphoribohydrolase (Kurakawa *et al.*, 2007; Osugi and Sakakibara, 2015). Studies of growth phenotypes and stable isotope-labeling experiments using the higher order T-DNA insertion mutants (e.g. *log1log2log3log4log5log7log8*) in Arabidopsis have demonstrated the central role of the LOG-mediated pathway in cytokinin activation for normal growth and development, including lateral root formation and root and shoot morphology (Kuroha *et al.*, 2009; Tokunaga *et al.*, 2012). However, the existence and physiological importance of a LOG-independent pathway involving sequential dephosphorylation and deribosylation remained elusive. Recently, the gene encoding cytokinin/purine riboside nucleosidase 1 (CPN1) was identified through phytohormone profiling in rice cultivars (Kojima *et al.*, 2023). CPN1 catalyzes the deribosylation of cytokinin nucleoside precursors and other purine nucleosides. Notably, CPN1 localizes to the cell wall and is implicated in apoplastic cytokinin metabolism (Fig. 2). Loss-of-function mutants of CPN1 display diminished expression of cytokinin marker genes in response to *t*Z riboside (*t*ZR), indicating its involvement in converting riboside precursors transported through the xylem into their active form within the leaf apoplastic space. This localization can enable efficient ligand supply to cytokinin receptors at the plasma membrane (Antoniadi *et al.*, 2020; Kubiasová *et al.*, 2020). The growth phenotype of CPN1 mutants is less pronounced compared to LOG mutants, and the translocation of *t*ZR is influenced by nitrogen availability, suggesting that the CPN1-mediated LOG-independent pathway plays a role in cytokinin supply for environmentally responsive modulation of plant growth. In Arabidopsis, the ortholog of CPN1, known as nucleoside hydrolase 3 (NSH3), is involved in extracellular ATP metabolism, and its expression is upregulated in response to disease-defense signals like jasmonic acid (Jung *et al.*, 2011; Daumann *et al.*, 2015). Similar expression patterns observed in rice suggest that CPN1 possesses multiple functional roles.

The genetic entity of the nucleotidase responsible for the LOG-independent pathway remained elusive for a long time. Enzymatic characterization of partially purified preparations showed a neutral pH optimum, suggesting an intracellular localization (Chen and Kristopeit, 1981). Interestingly, a member of the LOG protein family in rice, LOGL5 (GY3), was recently reported to function as a 5'-ribonucleotide phosphohydrolase rather than as a phosphoribohydrolase (Wu *et al.*, 2023). Results from studies of CPN1 and GY3 in rice suggest that the LOG-independent pathway consists of two reactions that occur at spatially distinct locations. Considering that nucleosides taken up by the cell are immediately phosphorylated (Tokunaga *et al.*, 2012) and, conversely, nucleotides released from the cell are immediately dephosphorylated by extracellular acid phosphatases, then the metabolic link between GY3 and CPN1 would be relatively small. Therefore, the efficiency of the successive reactions of each enzyme to produce an active form is considerably lower than that of LOG. While CPN1 plays a role in activating cytokinin precursors transported through the xylem (Kojima *et al.*, 2023), GY3 may be involved in controlling ribotide and riboside levels by coupling with adenosine kinases within cells (Fig. 2). This model was validated by the observation that higher-order Arabidopsis *LOG* mutants accumulate both nucleotide-type precursors and nucleoside-type precursors (Tokunaga *et al.*, 2012). The possibility of isoenzymes with 5´-phosphohydrolase activity has not yet been explored for Arabidopsis LOG homologs. Further investigations are needed to address the universality of *LOG* homolog involvement in the LOG-independent pathway in various plant species.

## Question 4. Is *cis-*hydroxylation involved in the biosynthetic pathway for *cis-*zeatin?

Both *t*Z and iP are synthesized by IPT and have higher cytokinin activity than *c*Z in various plant species, including Arabidopsis and rice (Stolz *et al.*, 2011; Choi *et al.*, 2012; Kiba *et al.*, 2013, 2023; Osugi *et al.*, 2017). The *c*Z form is thought to be produced by tRNA modification by tRNA-isopentenyltransferase (tRNA-IPT) following tRNA turnover (Fig. 1). In fact, the *c*Z-type species is almost absent from Arabidopsis tRNA-IPT double mutants (*atipt2atipt9*) (Miyawaki *et al.*, 2006). The step in which a hydroxyl group is introduced at the *cis*-position of *c*Z species is poorly understood in plants. In *Escherichia coli*, a nonheme di-iron monooxygenase, a *miaE* gene product, catalyzes the hydroxylation reaction at the *cis*-position (Buck and Ames, 1984; Corder *et al.*, 2013), but no homologous candidate gene has been found in plants. Among Arabidopsis *atipt2* and *atipt9* mutants, *atipt2* significantly reduces *c*Z endogenous production (Nguyen *et al.*, 2023). The subcellular localization of AtIPT2 and AtIPT9 has not been experimentally verified, but AtIPT2 encodes eukaryotic-type tRNA-IPT, suggesting that it is involved in cytoplasmic tRNA modification. This hypothesis is consistent with previous findings that the hydroxylase acting on the *cis-*position uses prenyl groups from the mevalonic acid pathway as a substrate (Kasahara *et al.*, 2004). It is possible the *cis-*hydroxylation does not occur after prenylation of tRNA but before, namely by *cis-*hydroxylation of DMAPP followed by prenylation. A comprehensive gene search for the hydroxylase is needed to answer this critical question.

As for the isomerization between *t*Z and *c*Z, studies have yet to identify the enzyme responsible for the conversion (Hluska *et al.*, 2017; Kudo *et al.*, 2012).

Cytokinin endogenous levels have been profiled in diverse plant species, collectively showing that *c*Z-type cytokinins accumulate in large amounts in a variety of plant species (Gajdošová *et al.*, 2011). Considering that most of the accumulated *c*Z molecules are glucosides (Gajdošová *et al.*, 2011; Osugi and Sakakibara, 2015), perhaps uridine diphosphate-dependent glycosyltransferases (UGTs) preferentially serve as substrates for *c*Z in these plant species stabilize *c*Z produced from tRNA turnover by glycosylation and increase its accumulation.

## Question 5. How does Tmr predominantly use HMBDP as a substrate *in vivo*?

Some plant pathogenic bacteria manipulate the fate of host plant cells by producing cytokinins to create a favorable environment for their survival. For instance, *Fusarium pseudograminearum* (Sørensen *et al.*, 2018) and *Rhodococcus fascians* (Radhika *et al.*, 2015) synthesize unique cytokinins. Similarly, *Agrobacterium tumefaciens* generates substantial amounts of *t*Z and indole-3-acetic acid (Palni *et al.*, 1983; Morris, 1986; Ueda *et al.*, 2012) by integrating the T-DNA region of the Ti-plasmid into the host cell nuclear genome, although some aspects of this process remain unclear, including the translocation mechanism of Tmr into the plastid. Despite lacking a typical transit peptide region, Tmr from *A. tumefaciens* localizes to the plastid stroma of the host plant cell (Sakakibara *et al.*, 2005). It remains to be determined whether Tmr is imported to the plastids by the canonical Toc-Tic system (Nakai, 2018; Rochaix, 2022) or by another system, such as membrane traffic through the Golgi apparatus (Villarejo *et al.*, 2005; Kitajima *et al.*, 2009; Bellucci *et al.*, 2017). Another intriguing aspect is the selective utilization of substrates in host plant cells. Where *in vitro* analyses show that Tmr exhibits similar affinities and reaction efficiencies toward the substrates DMAPP and (*E*)-4-Hydroxy-3-methyl-but-2-enyl diphosphate (HMBDP) (Sakakibara *et al.*, 2005; Sugawara *et al.*, 2008), *in planta* expression of Tmr results in the exclusive use of HMBDP to synthesize significant amounts of *t*Z (Sakakibara *et al.*, 2005; Ueda *et al.*, 2012). Since both substrates are present in the plastid stroma, it is reasonable to propose that Tmr can potentially use both.

In the methylerythritol phosphate (MEP) pathway, the ratio of isopentenyl diphosphate to DMAPP synthesized from HMBDP is approximately 5:1 (Rohdich *et al.*, 2002), and subsequent isomerization equalizes the ratio between these two molecules (Rodríguez-Concepción and Boronat, 2015; Gutensohn *et al.*, 2022). Although the precise *in vivo* ratio of HMBDP to DMAPP remains unknown, it is unlikely that the substrate concentration ratio alone explains the preferential accumulation of *t*Z. This is evident from the fact that artificially adding a transit peptide to another *Agrobacterium* IPT named *trans-*zeatin secretion (Tzs), which shares enzymatic properties with Tmr (Sugawara *et al.*, 2008), does not lead to the preferential accumulation of *t*Z-type species in the plastid, but instead results in the accumulation of iP-type cytokinins (Ueda *et al.*, 2012). Another possibility is that Tmr interacts with ispG, the HMBDP-generating enzyme in the MEP pathway (Hecht *et al.*, 2001), thus facilitating substrate channeling. It is also conceivable that the spatial distribution of prenyl-donor substrates and Tmr proteins within the plastid may be heterogeneous, leading to variations in their local concentrations. This possibility might result in a relatively higher concentration of HMBDP surrounding Tmr.

## Conclusions and perspectives

This review has focused on the cytokinin metabolic system, especially the steps for the biosynthesis of iP, tZ, and cZ, highlighting still unresolved points and suggesting possible processes. Although not discussed in this review, DZ, which is often abundant in legumes, is a biologically stable species shown to be derived from tZ (Letham and Palni, 1983; Nandi *et al.*, 1988; Martin *et al.*, 1989; Gaudinová *et al.*, 2005). However, the intracellular compartment where the synthesis reaction takes place and the gene(s) encoding zeatin reductase have not been identified.

To fully understand the biosynthetic pathway of cytokinins, it will be necessary to characterize the entire metabolic system, including not only the substance conversion reactions, but also the mode of transport across membranes. Although several genes involved in the influx and efflux transport of cytokinins have been recently reported, few studies have been conducted on the transporters that are fundamental to the biosynthetic process. The same is true for other phytohormones that are biosynthesized through multiple intracellular compartments. Future efforts to understand the metabolic system, including the spatial axis at the subcellular level, will be essential.

## Supplementary data

The following supplementary data are available at *JXB* online.

Fig. S1. A current view of subcellular compartmentation of cytokinin metabolism and transport processes.

erae348_suppl_Supplementary_Figure_S1
