# Supplementary material for: Five unaddressed questions about cytokinin biosynthesis
Source: J Exp Bot. 2024 Sep 19;76(7):1941–9. doi: 10.1093/jxb/erae348 (PMC12066119; doi:10.1093/jxb/erae348)
Supplement: erae348_suppl_Supplementary_Figure_S1 [file erae348_suppl_supplementary_figure_s1.pdf]

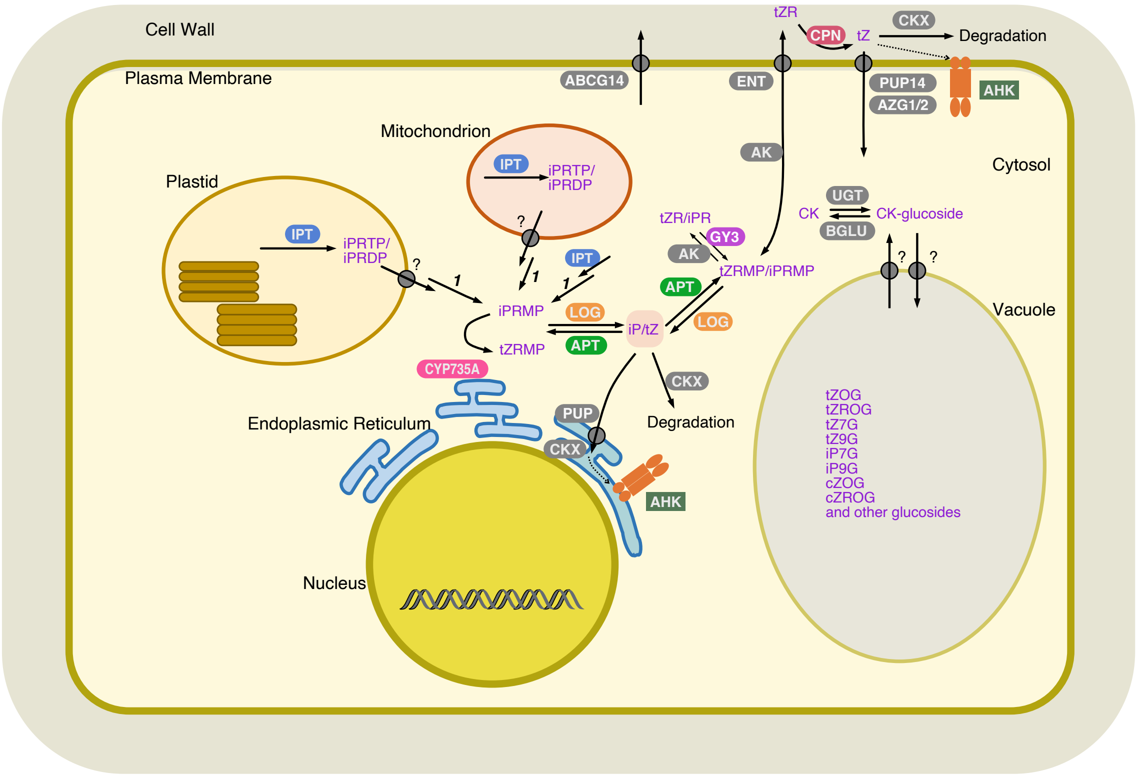

Supplementary Figure S1. A current view of subcellular compartmentation of cytokinin metabolism and transport processes.

Solid arrows represent metabolic flow, and the dashed line represents recognition by cytokinin sensory histidine kinase AHK. ***1***, phosphatase; ?, indicates an unidentified transporter; ABCG14, ATP-binding cassette transporter subfamily G14; AK, adenosine kinase; APT, adenine phosphoribosyltransferase; AZG, AZA-guanine resistance; BGLU, β-glucosidase; CPN, cytokinin/purine riboside nucleosidase; CKX, cytokinin oxidase; CYP735A, cytochrome P450 monooxygenase 735A; ENT, equilibrative nucleoside transporter; GY3, 5'-ribonucleotide phosphohydrolase grain yield 3; IPT, adenosine phosphate-isopentenyltransferase; LOG, cytokinin riboside 5'-monophosphate phosphoribohydrolase lonely guy; PUP14, purine permease 14; UGT, UDP-glycosyltransferase.
